# Bioactivity determination of methanol and water extracts for roots and leaves of Kenyan *Psidium guajava L* landraces against pathogenic bacteria

**DOI:** 10.1186/2193-1801-2-670

**Published:** 2013-12-13

**Authors:** Mercy Liharaka Kidaha, Amos Emitati Alakonya, Aggrey Benard Nyende

**Affiliations:** Institute for Biotechnology research, Jomo Kenyatta University of Agriculture and Technology, P.O BOX 62000-00200, Nairobi, Kenya

**Keywords:** Landraces, *Psidium guajava*, *Staphylococcus aureus*, *Bacillus subtilis*, *Escherichia coli*

## Abstract

Guava (*Psidium guajava L*) is native to South America and exists as both wild and cultivated. Guava has been used as a source of food and raw materials for pharmaceuticals. The aim of this study was to determine bioactivity of methanol and water extracts from root and leaves of Kenyan guava landraces against selected pathogenic bacteria. Study samples were collected from Western and South Coast of Kenya. One hundred grams of leaf and root ground powders were used for sequential extraction using methanol and water. Extracts were evaporated and 0.2gms dissolved using the extraction solvent and tested against gram positive (*Staphylococcus aureus, Bacillus subtilis*) and negative bacteria *(Escherichia coli)*. Data on inhibition zone was taken in mm and analyzed at 95% confidence interval. Extracts from Western region had significant inhibition compared to Coastal region. The two regions have different climatic conditions that result in these plants having different compounds even though they are the same species. Roots had higher inhibition compared to the leaves as they contain high levels of tannins compared to leaves. Water as an extracting solvent had higher inhibition than methanol as it is more polar and it absorbs more bioactive compounds. *S. aureus* was most inhibited followed by *E. coli* and *B. subtilis* respectively. There was no significant difference between the gram positive and negative bacteria. Remarkably, some methanol and water root extracts had significant inhibition against bacteria when compared to some commercial antibiotics used. Results of this study indicate that Kenyan guava roots from Western Kenya extracted with methanol and water have a potential to be used as a source of active compounds in treatment of gram positive and gram negative bacteria pathogens.

## Introduction

Guava (*Psidium guajava L*) is native to South America where it exists in both wild and cultivated form. In East Africa, guava grows well from sea level to an altitude of about 2,000 m above the sea level. The tree generally begins bearing 1 or 2 years after planting and continues fruiting for 30 years (Beentje, 1994). The fruit is an excellent source of vitamin C, calcium, potassium and iron (Valdes-Infante et al., 2007). It is consumed as ripe or processed into juices and the leaves used in traditional therapy of dysentery and diabetes.

Resistance has increased as some bacteria change in ways that reduce or eliminates the efficiency of drugs or chemicals or other curative and preventive agents. The bacteria survive and multiply causing more harm. The bacteria cause harm by neutralizing the antibiotic before its efficiency, others rapidly pump the antibiotic out, others change antibiotic attack site so that it cannot affect the function of bacteria (CDC, 2012).

Guava leaves and barks have a long history of medicinal uses that are still in use today. In Peruvian guava herbal medicine are employed to treat diarrhoea, vomiting, coughs and virginal discharges. Currently the leaves are used to treat diarrhoea in latin America, central and western Africa (Gutierez et al., 2008). Guava has chemical components present that make it more effective as medicinal herb such as tannins, phenols, tritepenes flavonoids, saponnins, carotenoids, lectins, vitamins, fibre and fatty acids (Begum et al., 2002). Isolated compounds from guava leave with herbal effect are quercetin, morin quarcertin-3-0-glucopyranoside guaijavarine and quercetin is the most active compound (Arima and Danno, 2002,2007). The Guava seed contains crude compounds of 11.52% proteins, 0.54% oil and 79.62% crude fibre (Gamal et al., 2011). Herbal history of guava has led to modern study of guava extracts. Its herbal use to treat various diseases has been validated in various chemical studies. A plant drug has been developed from the leaves for the treatment of acute diarrhoea and showed to be effective in treating adults. Guava leaves and fruit juice has also been tested in treatment of infantile diarrhoea and the results showed that, those who were treated with guava recovered at 3 days which was shorter than the controls and the study concluded that guava had good curative effect on infantile rotoviral enteritis (Wei et al., 2000).

An alcoholic leaf extract has also been reported to have morphine like effect by inhibiting gastrointerstinal release of chemicals in acute diarrhoea. The morphine like effect is related to quercetin. Lectin compounds of guava have been shown to bind to *E. coli* which is a common causative agent of diarrhoea and prevents adhesion to intestinal wall hence preventing diarrhoea infection (Coutino et al., 2001). Bark and leaf extracts have shown to have in vitro toxic action against numerous bacteria. In studies guava leaf and bark have shown activity against *S. aureus. Shigella, Salmonella, Bacillus* and *E. coli* that are causative agents to diarrhoea (Mohammad et al., 2011; Doughari and Manzara, 2008). In vivo studies to find out the treatment of serum content of hepatoxic rats it was reported that ethanolic extracts of guava and pomegaranate significantly increased serum albumin content compared to carbon tetrachloride (Mohieiden et al., 2011). In similar studies ethanolic extracts of guava and pomegranates significantly reduced the liver weight of hepatoxic rats compared to carbon tetrachloride (Mohieiden et al., 2011). Studies have also shown antifungal, anti-yeast, ant amebic and antimalarial functions (Somwyas et al., 2013). A study in 2003 with guinea pigs Brazillan researcher demonstrated that leaf extracts have various effects on cardiovascular system which may be beneficial in treating irregular arrhythmia (Yamashiro et al., 2003). Guava leaf provided antioxidant that protects the heart and improved d myocardial function. In two randomized study the consumption of guava for 12 weeks showed reduced blood pressure by an average of 8 points, decreased total cholesterol by 9% decreased triglycerides by 8%

The guava has potential bioactive compounds that can be used for medicinal purposes. The aim of this study was to determine the activity of methanolic and water leaf and root extracts of Kenyan guava landraces against pathogenic bacteria. Hypothesis of the study is that extracts from Kenyan guava landraces do not have bioactivity against the bacterial pathogens. Studies on bioactive compounds of Kenyan guava have not been carried out and this has limited its exploitation for pharmaceutical products and as food.

## Materials and methods

### Study site and sampling

**Figure 1:**
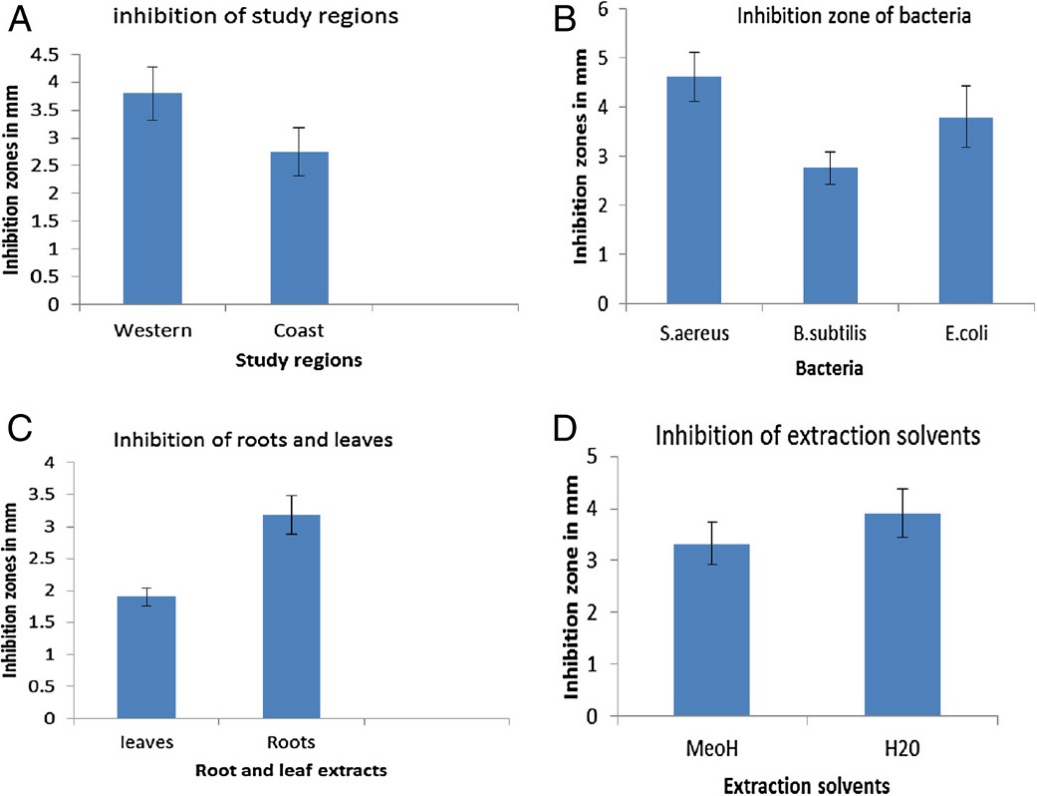
**Relative inhibition rate of extracts based on study regions, pathogen evaluated, plant tissue used and solvent used.**

Samples were collected from 9 sites; 6 sites from western Kenya in Butere Mumias district and three sites from Coastal region. Western sites included Kisa, Evukambuli, Bumamu, Bukura, Makunga and Sabatia. Temperatures in western ranges from 17˚C-22˚C. Coastal sites included; Shimba Ukunda and Msambweni. Coastal sites have an annual temperature of 24.2˚C (Figure 1). Purposive sampling technique was used and the trees under study were tagged. An average of 6 roots and 6 leaf samples of 100 gms each, were collected from each site. Samples from Kisa were denoted as SHIS001-006. Evukambuli samples were labeled as EVUK001-006. Bumamu samples were labeled as BUM001-006. Bukura samples were labeled as BUK001-009. Makunga samples were labeled as MAK001-006. Sabatia samples were labeled as SAB001-SAB01. Shimba samples were labeled as SHIM001-006.Ukunda samples were labeled as UK001-004. Msambweni samples were labeled as MSA001-005. Samples were harvested, cleaned with tap water, dried under shed and then ground to powder using a laboratory mill.

### Bioactivity assay

#### Micro-organism and standard antibiotics

Micro-organisms used were obtained from National Public Health Laboratories Kenya. Pure cultures of *B. subtilis, E. coli* and *S. aereus* were cultured on nutrient agar 28 g/l. Antibiotic discs octodisks (Himedia Laboratories Ltd KGL2/45) impregnated with standard antibiotics; ampicillin (Amp), tetracycline (Te), centramaxazole (COT), streptomycin (S), kanamycin (K), gentamicin (Gen), sulphamohoxazole (Sx) and chloromphenical (C) were used as positive control while distilled water and methanol were used as a negative control.

#### Extraction of bioactive compounds and screening for bioactivity

Five grams of each sample was weighed and dissolved in 30 ml of solvents. This was extracted sequentially using methanol then followed by water as described by Uthayasara et al., (2010). The extracts were dried using rotary evaporator at 30-40°C and 0.2 gms weighed then dissolved in 1 ml of each solvent and stored at 4°C. Paper discs were soaked in 20 μL of the extract, allowed to evaporate and then inoculated on the plates with cultured bacteria this was done in 3 replicates. This was done alongside with antibiotic discs as positive control and water as a negative control. Data on the inhibition zones was taken 24 hrs after inoculation and recorded in millimeters.

## Results

Plant roots and leaves are considered to be active against micro-organisms when the inhibition zone is greater than 6 mm (Saadabi and Ayoub, 2009). In this study, most extracts showed inhibition against the micro-organism but only a few of them had inhibitions above 6 mm.

### Inhibition of *S. aureus*with methanol and water root and leaf extracts of guava from Coastal and Western Kenya

Leaves that inhibited *S. aureus* when extracted using methanol included BUM003, BUM004, BUM005, and BUM006 and MSA003 giving 9.3% of western landraces and 6.7% of Coastal landraces. Roots inhibition against *S. aureus* was observed in; SAB002, SAB006, EVUK001, EVUK002, MSA001, UK002, UK003 which is 13.95% of Western landraces and 6.7% of Coastal landraces. Leaves that showed inhibition against *S. aureus* when extracted using water included BUK008, BUK009 which is 4.6% of Western landraces and none from Coastal was inhibited. Roots extracted using water that inhibited the growth of *S. aureus* was observed in; EVUK005, EVUK006, SAB009, SAB01, SHIS001, BUK003 and BUK004 representing 3.95% of Western landraces and none from Coastal landraces*.* When methanol root extracts are compared to the commercial antibiotics (p = 0.978) they were not significantly different however UKU002, EVUK001 and EVUK002 were significantly different when compared K, Gen and COT (Table 1). When water root extracts were compared to the commercial antibiotics at (p = 0.848) they were not significantly different however EVUK005, EVUK006 and SHIS001 root extracts were significantly different when compared to K, Gen and COT (Table 1).

**Table 1:** **Inhibition of** ***S. aureus*** **with methanol and water root and leaf extracts of guava from Coastal and Western Kenya compared to commercial antibiotics**

	Methanol extracts in mm			Water extracts in mm	
Samples	Roots	Leaves	Samples	Roots	Leaves
MSA001	1.67 ± 0.33	0	EVUK005	10.67 ± 0.67	0
MSA003	0	4.67 ± 0.33	EVUK006	10.33 ± 0.88	0
UKU002	13.33 ± 1.67	0	SAB009	5.33 ± 0.33	0
UKU003	2.17 ± 0.17	0	SAB01	5.67 ± 0.33	0
EVUK001	9.67 ± 1.86	0	SHIS001	10.67 ± 1.20	0
EVUK002	13.33 ± 1.67	0	BUK003	5.33 ± 0.88	0
SAB002	3.67 ± 0.33	0	BUK004	3.67 ± 0.33	0
SAB006	1.17 ± 0.44	0	BUK008	0	0.50 ± 0
BUM003	0	2.33 ± 0.33	BUK009	0	1.67 ± 0.33
BUM004	0	2.00 ± 0	Water	0	0
BUM005	0	1.00 ± 0			
BUM006	0	1.00 ± 0			
Methanol	0	0			
Positive controls					
S	12.33 ± 0.88		C	12.83 ± 0.44	
K	8.67 ± 0.33		Amp	12.83 ± 0.44	
Gen	8.67 ± 0.33		TE	22.5 ± 0.28	
Sx	8.67 ± 0.33		COT	4.17 ± 0.60	

### Inhibition of *B. subtilis*with methanol and water root and leaf extracts of guava from Coastal and Western Kenya compared to commercial antibiotics

Methanol root extract that inhibited the growth of *B. subtilis* included SAB003, SAB007, SHIS001-SHIS004 which is 16.98% of Western landraces and none from Coastal landraces. Methanol leaf extracts were not active against *B. subtilis*. Inhibition of water leaf extracts against *B. subtilis* was observed in MSA004 and MSA005 (13.3% of Coastal landraces), SAB001, SAB006, SAB009, SAB01, BUM001, BUM002, BUM005, BUK001, BUK002, EVUK006, SHIS001, SHIS002, SHIS003, representing 30.23% of Western landraces. When methanol extracts are compared with commercial antibiotics they were not significantly different (p = 0.999) however SHIS002 root extract was significantly different when compared to S, K, Gen, Amp, C, COT and Sx. When water root extracts are compared to the commercial antibiotics they were not significantly different (p = 0.976) however SAB001 and SAB002 root extracts were significantly different when compared to S, K, Gen, Sx, C and Amp while SAB007 root extract was significantly different when compared to Sx, C and Amp (Table 2).

**Table 2:** **Inhibition of** ***B. subtilis*** **with methanol and water root and leaf extracts of guava from Coastal and Western Kenya compared to commercial antibiotics**

	Methanol extracts in mm			Water extracts in mm	
Samples	Roots	Leaves	Samples	Roots	Leaves
SAB003	0.83 ± 0.33	0	SAB008	3.33 ± 0.33	0
SAB007	0.83 ± 0.17	0	SAB001	0	0.67 ± 0.17
SHIS001	3.67 ± 0.33	0	SAB006	0	2.33 ± 0.33
SHIS002	9.33 ± 0.33	0	SAB009	0	0.67 ± 0.16
SHIS003	3.67 ± 1.33	0	SAB01	0	0.67 ± 0.17
SHIS004	4.67 ± 0.88	0	BUM001	0	2.33 ± 0.33
Methanol	0	0	BUM002	0	2.33 ± 0.33
**Positive control**			BUM005	0	2.33 ± 0.33
S	6.67 ± 0.33		BUK001	0	1.17 ± 0.16
K	5.83 ± 0.60		BUK002	0	1.33 ± 0.33
Gen	5.67 ± 0.33		EVUK006	0	1.17 ± 0.16
Sx	4.67 ± 0.6		SAB001	10.67 ± 2.33	0
C	2.83 ± 0.17		SAB002	12.67 ± 0.33	0
Amp	4.67 ± 0.33		SAB007	7.33 ± 0.33	0
TE	20.33 ± 0.33		Water	0	0

### Inhibition of *E. coli*with methanol and water root and leaf extracts of guava from Coastal and Western Kenya compared to commercial antibiotics

Methanol leaf extracts were not active against *E. coli*. Inhibitions were only observed in the root extracts of EVUK002, EVUK003, EVUK005, SHIS001, SHIS002, SHIS003 and SHIS004 representing 16.98% of Western landraces. Coastal extracts were not active against *E. coli* Inhibition in water leaf extracts were only present in MAK003 and MAK004 representing 4.6% of Western collections and Coastal extracts were not active against *E. coli*. Water root extracts that inhibited *E. coli* included MSA003, MSA004, MSA005 representing 20% of Coastal collections whereas active samples from Western included; EVUK002, SAB001, SAB003, SAB004, SAB005, SAB006, SAB007, SAB008 and SAB009 resulting in 20.9% of Western collections. Methanol root extracts are not significantly different (p = 0.868) when compared to commercial antibiotics but SHIS004 root extract was significantly different when compared to Gen. Water root extracts are not significantly different (p = 0.139) when compared to the commercial antibiotics, but SAB005 root extract is significantly different when compared to S, K, Gen, COT, Amp and SX (Table 3).

**Table 3:** **Inhibition of** ***E. coli*** **with methanol and water root and leaf extracts of guava from Coastal and Western Kenya compared to commercial antibiotics**

	Methanol extracts in mm			Water extracts in mm	
Samples	Roots	Leaves	Samples	Roots	Leaves
EVUK002	2.33 ± 0.33	0	MSA003	4.50 ± 1.02	0
EVUK003	2.33 ± 0.882	0	MSA004	7.50 ± 1.90	0
EVUK005	1.83 ± 0.167	0	MSA005	1.00 ± 0	0
SHIS001	1.67 ± 0.33	0	EVUK002	2.67 ± 0.67	0
SHIS002	1.33 ± 0.333	0	SAB001	4.67 ± 0.88	0
SHIS003	3.33 ± 0.333	0	SAB003	0.50 ± 0	0
SHIS004	6.67 ± 0.667	0	SAB004	7.00 ± 0.57	0
Methanol	0	0	SAB005	11.33 ± 0.88	0
**Positive controls**			SAB006	4.67 ± 1.20	0
S	9.67 ± 0.3		SAB007	2.00 ± 0.57	0
K	8.33 ± 0.67		SAB008	2.17 ± 0.60	0
Gen	6.33 ± 0.24		SAB009	3.33 ± 1.20	0
Sx	8.00 ± 0.57		MAK003	0	4.67 ± 0.33
C	11.67 ± 0.33		MAK004	0	4.33 ± 0.33
Amp	7.50 ± 0.29		Water	0	0
TE	14.0 ± 1.15				
COT	9.67 ± 0.67				

### Efficiency of inhibition

The average inhibition among positive samples was highest among extracts collected from Western Kenya followed by Coastal region (Figure 1a). *S. aureus* was the most inhibited pathogen followed by *E. coli* and *B. subtilis* respectively (Figure 1b). Root extracts showed higher inhibition zones than leaves (Figure 1c). Water as an extracting solvent had higher inhibition zone than methanol (Figure 1d).

## Discussion

Extracts collected from Western Kenya had higher inhibition compared to those collected in the south Coast region Kenya. Different regions have different climatic conditions ranging from soil type, rainfall availability, temperature and humidity that result in these plants having different compounds even though they are the same species. Different parts of guava plant have potential to treat different types of diseases caused by bacteria, fungi, plasmodium (Gutierez et al., 2008). This different parts contain different bioactive compounds that are effective against micro-organisms (Hsieh et al. 2007). Micro-organisms growths are inhibited by essential oils in leaves that are involved in different modes of action and may be due to hydrophobicity. The essential oils have a lipid bilayer that renders them more permeable leading to leakage of cell membrane (Hsieh et al. 2007). The antimicrobial effect of guava leaves tested positive against *S. aureus*, *E. coli* and *B. subtilis*. The essential oils in the leaves enhanced the inhibition against the micro-organism (Goncalves et al., 2005).

Root extracts in this study had greater inhibition against the micro-organism and this is due to the high levels of tannins present in the roots (Jain et al., 2003). In recent years tannins have received great popularity, seemingly consumption of foods with high levels of tannins has a great potential in treatment of most ailments. Their activity against bacteria may be related to their ability to inactivate microbial adhesions, enzymes and cells envelop that transport proteins (Cowan, 1999). In most studies done leaves are the more preferred compared for analysis than roots. Harvesting of leaves will not affect the growth of the plant compared to other parts which are crucial in plant physiological processes.

Extraction solvents differed in the rates of their activity methanol extracts had less inhibition compared to water. In previous studies methanolic extracts suppressed the growth of *S. aureus* and *E. coli* (Doughari and Manzara, 2008). Ethanol extracts isolated flavonoid compounds that were effective against *S. aureus* and *E. coli*(Metwally, 2010). Contrary results on the activity of methanolic leaf extracts was reported by (Gaidem et al., 2010) where by the extracts had no effect on the growth *E. coli, S. aureus* and *S. fecalis*.

There was no great difference in the inhibition between gram positive and gram negative bacteria as all were inhibited (p = 0.578). *S. aureus* growth was inhibited by methanolic leaf and root extracts (Doughari and Manzara, 2008). This extracts could be used in the prevention of food poisoning diseases, wounds as they are caused by *S. aureus* or in the treatment of multi resistance strains of *S. aureus*. Sanches et al., (2005) showed that a combination of ethanol and water as extraction solvent with the roots, leaves stems and barks had ability to inhibit only gram positive bacteria as compared to aqueous extracts. All the gram negative bacteria were not inhibited which is contrary to the results where methanol and water root and leaf extracts had activity against both gram negative and gram positive bacteria. Methanol leaf extracts showed significant protection against *S. aureus* and *E. coli* (Mohammed et al., 2012). Water extracts had more active compounds than methanol extracts (Esmonel et al., 2012) and that activity of water may be associated with the common practice in traditional medicine to use plant extracts prepared in the form of infusion and decoctions. In the study by (Esmonel et al., 2012) methanol leaf extracts inhibited the growth of 5 strains of *S. aureus* while water inhibited 7 strains of *S. aureus*. These results are similar to the results by Saadabi and Ayoub, (2009) where the most polar solvent extracted many active compounds. In a related study contradicting results were reported where methanol leaf extracts had greater inhibitions than aqueous leaf extracts (Anas et al., 2008).

In this study it was noted that commercial antibiotics showed a higher inhibitory activity than most extracts. Some extracts demonstrated higher activity than some antibiotics against gram positive and gram negative bacteria which shows the broad spectrum activity of guava extracts. This gives the guava great potential to be used as a source of antibiotic for drug development against bacterial infections, but it limits the use of guava from the two regions as a source in development of more potent drugs against this bacteria.

In conclusion guava leaf and root extracts have activity against bacteria pathogens and the test organisms were susceptible to commercial antibiotics.
